# A single-center, two-year, retrospective study of epidemiological characteristics of respiratory tract infections in Changzhou, China

**DOI:** 10.3389/fpubh.2026.1828799

**Published:** 2026-06-05

**Authors:** Yan Wang, Hongxing Zhou, Tongbao Feng, Ping Zhang

**Affiliations:** Department of Clinical Laboratory, The Second People's Hospital of Changzhou, The Third Affiliated Hospital of Nanjing Medical University, Changzhou, China

**Keywords:** epidemiological analysis, *Mycoplasma pneumoniae* infection, real-time polymerase chain reaction, respiratory pathogens, respiratory tract infections, viral infection

## Abstract

**Introduction:**

This was a single-center, retrospective study that analyzed the epidemiological characteristics of respiratory tract infections (RTIs) in the Second People’s Hospital of Changzhou from December 2023 to November 2025.

**Methods:**

Briefly, pharyngeal swabs from 24,380 cases were harvested and subjected to RT-PCR to detect influenza A and B viruses (Flu A and B), adenovirus (ADV), human rhinovirus (HRV), respiratory syncytial virus (RSV), and *Mycoplasma pneumoniae* (MP).

**Results:**

The total positive rate is 47.00% (11,459/24380). HRV (13.27%) was the predominant pathogen for RTIs, and HRV combined with ADV and other pathogens primarily accounted for co-infections. The overall detection rate of RTIs showed a significant sex-related relevance (χ^2^ = 17.606, *p* < 0.001), and the positivity for each of the six respiratory pathogens differed across age groups (*p* < 0.001). Individuals at 12-17 years (11.97%), 18-45 years (6.83%), 6-11 years (24.14%), <1 year (22.35%), 3-5 years (21.36%), and 6-11 years (27.48%) were more likely to be affected by FluA, FluB, ADV, RSV, HRV, and MP, respectively. In our center, FluA caused seasonal peaks in winter and autumn, and HRV activity peaked in autumn and spring. Infections by the remaining respiratory pathogens were sporadic. Additionally, outpatients were more likely to be affected by RTIs than inpatients. However, this relationship may be reversed for RSV infection and MP infection as age was considered.

**Conclusion:**

In conclusion, RTIs caused by the six common respiratory pathogens in this local institution were associated with sex, age, seasonality and patient status.

## 1 Introduction

Respiratory tract infections (RTIs), a severe public health concern, cause four million deaths worldwide each year (1), and inflict higher risks on populations of children, older adults, and individuals residing in underdeveloped regions (2). In China, the incidence of RTIs has elevated substantially (3). Viruses, bacteria, and *Mycoplasma* are common pathogens leading to RTIs (4, 5). Influenza A and B viruses (Flu A and B), adenovirus (ADV), human rhinovirus (HRV), respiratory syncytial virus (RSV), and *Mycoplasma pneumoniae* (MP) are prevalent respiratory pathogens with seasonal transmissions. Notably, severe RTIs like the COVID-19 pandemic greatly disrupt the socioeconomic cycle (6, 7).

A series of factors, such as region, population, and season, influence the epidemiologic pattern of RTIs and pathogen spectrum. In this single-center, two-year, retrospective study, we aimed to uncover the epidemiological characteristics of RTIs in the Second People’s Hospital of Changzhou (Changzhou, China) by detecting six common respiratory pathogens across subgroups via real-time polymerase chain reaction (RT-PCR). Our findings are expected to offer valuable epidemiological data to optimize public health policy and prevention strategies against RTIs in Changzhou.

## 2 Materials and methods

### 2.1 Study design

This was a single-center, two-year, retrospective study that analyzed the RT-PCR data of pharyngeal swabs from 24,380 cases between December 2023 and November 2025 in the Second People’s Hospital of Changzhou, Jiangsu Province, China. Jiangsu is a coastal province in East China, climatologically characterized by a subtropical monsoon pattern in the northern hemisphere with distinctive seasonal transitions from spring (March, April, and May), summer (June, July, and August), autumn (September, October, and November), to winter (December, January, and February). A waiver of informed consent was granted for the review of retrospective data. Study protocols were approved by the ethics committee.

### 2.2 Participants and retrospective data collection

**Figure 1:**
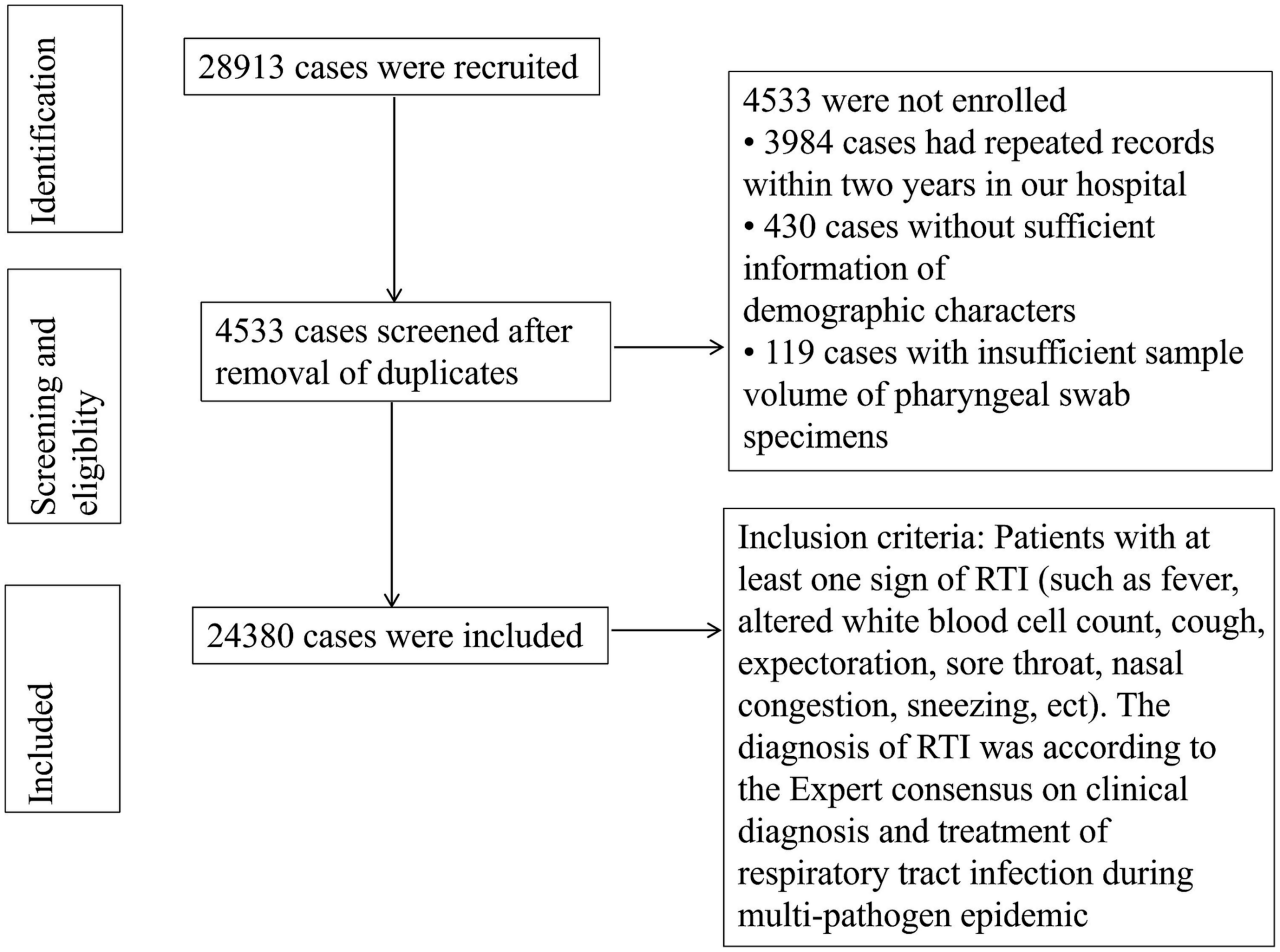
Flowchart presenting the steps of patient selection.

Patients manifesting at least one sign of RTIs (e.g., fever, abnormal white blood cell count, cough, expectoration, sore throat, nasal congestion, sneezing), regardless of its severity, were included. A diagnosis of RTIs was established according to the expert consensus (8). RT-PCR was carried out to detect respiratory pathogens in each participant. Excluded were those with missing clinical data or insufficient sample volume of pharyngeal swab specimens. Basic (sex, age), clinical characteristics and laboratory testing results were retrospectively collected. A flowchart showing patient selection is provided in Figure 1. This study was reported in accordance with the STROBE (Strengthening the Reporting of Observational Studies in Epidemiology) guidelines for cross-sectional/retrospective observational studies.

### 2.3 Collection of pharyngeal swabs

Sterile pharyngeal swabs, with an insertion depth of 7–8 cm and multiple rotations against the nasal wall, were collected by professional healthcare providers on the first or second day after admission. The specimens were immediately placed into a sterile tube containing normal saline, and sent to the clinical laboratory for testing. All specimens were timely detected, or stored at 4 °C for no more than 24 h.

### 2.4 RT-PCR for detecting respiratory pathogens

Nucleic acid was routinely extracted from pharyngeal swabs using a commercial kit (Sansure Biotech Inc., China) for RT-PCR of Flu A and B, ADV, HRV, and MP on a SLAN 96S PCR instrument (Hongshi, China). RT-PCR results were interpreted by the cycle threshold (Ct), and the positivity for respiratory pathogens was determined according to the recommended Ct thresholds. Lentiviral particles were used as positive control and internal standard, and normal saline as negative control. The following detection channels were assigned: FAM for FluA and ADV, HEX or VIC for FluB and HRV, and CY5 for RSV and MP. The ROX channel was used to detect the internal standard nucleic acid. The following criteria were simultaneously met. (1) Negative control: the ROX channel exhibited a distinct S-shaped amplification curve with a Ct value ≤40, while the FAM, HEX or VIC, and CY5 channels showed no amplification (No Ct) or a Ct value >40; (2) Positive control: the FAM, HEX or VIC, and CY5 channels displayed distinct S-shaped amplification curves with Ct values between 27 and 33, whereas the ROX channel showed no amplification (No Ct) or a Ct value >40. The positive case should display a distinct S-shaped amplification curve with a Ct value ≤40 for each pathogen.

### 2.5 Statistical analysis

All positive cases confirmed by RT-PCR were retrospectively identified. Presented in Excel 2024, clinical data were analyzed via Chi-squared or Fisher’s exact or Mann–Whitney test in SPSS 17.0 (SPSS Inc., Chicago, IL, United States). The multivariate logistic regression analysis was performed in SPSS 17.0, and the adjusted odds ratio (OR) with 95% confidence interval (CI) was used to determine the independent factor for RTIs after adjusting for age, sex, season and case type. *p* < 0.05 was defined to indicate significance.

## 3 Results

### 3.1 Baseline characteristics of participants

An overview of the selection of relevant studies is depicted in Figure 1. A total of 24,380 participants (7,968 outpatients and 16,412 inpatients) were retrospectively allocated, involving 13,314 (54.61%) male and 11,066 (45.39%) female individuals with a median age of 8 years (range: 3 days to 98 years) and a mean age of 29 ± 32 years. Age groups involved <1 year (infants, *n* = 1,432), 1–2 years (toddlers, *n* = 2,702), 3–5 years (preschool children, *n* = 4,518), 6–11 years (children, *n* = 5,190), 12–17 years (teenagers, *n* = 802), 18–45 years (young adults, *n* = 1,449), 45–65 years (middle-aged adults, *n* = 2,402), and >65 years (older adults, *n* = 5,885).

### 3.2 Positive cases of RTIs

**Figure 2:**
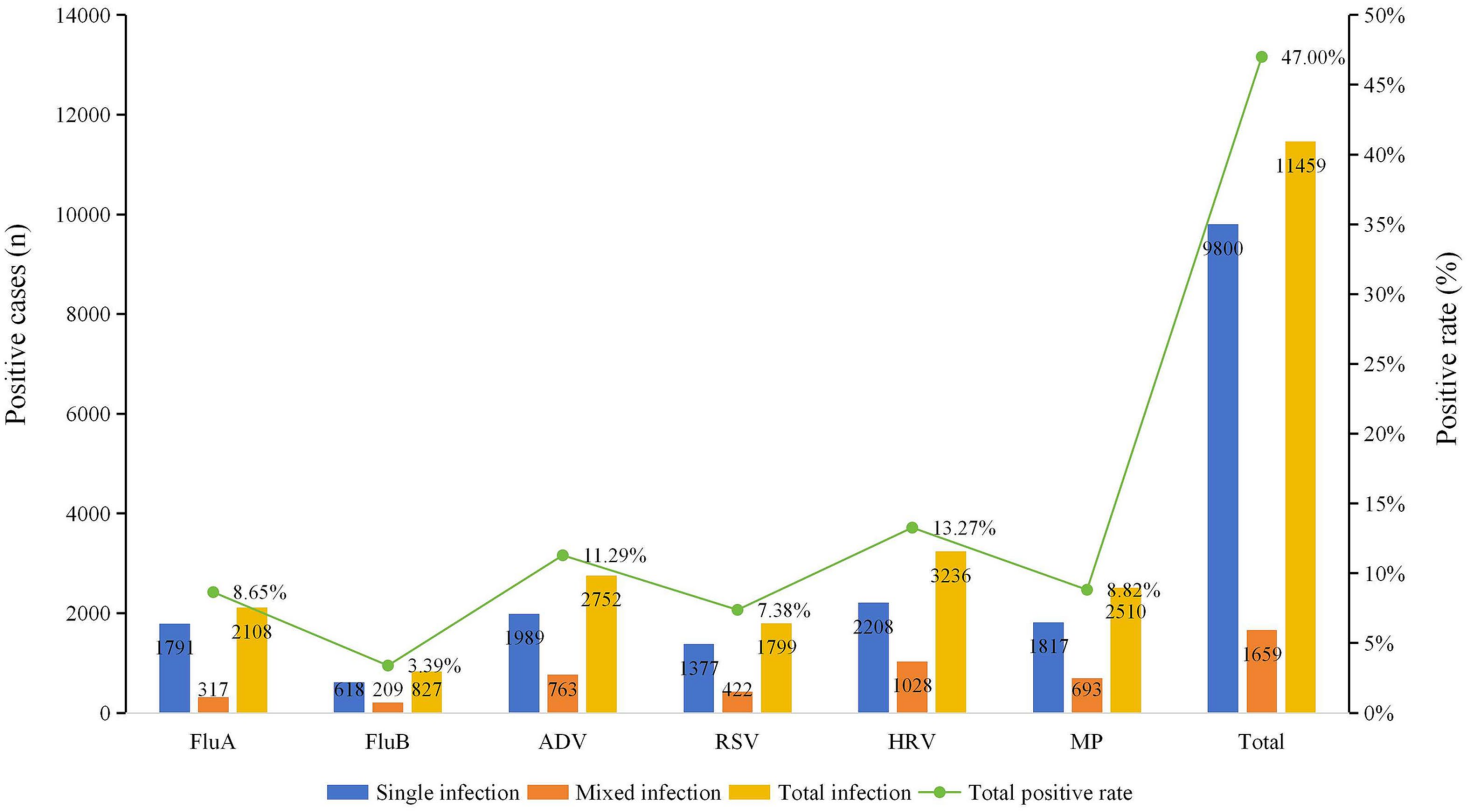
The positivity rate of respiratory pathogens in all participants.

Positivity for at least one tested respiratory pathogen was detected in 11,459/24,380 (47.00%) participants, including 9,800 (40.20%) single infections, and 1,659 (6.80%) co-infections. HRV (13.27%, *n* = 3,236) was the single and predominant pathogen leading to RTIs, followed by ADV (11.29%, *n* = 2,752), FluA (8.65%, *n* = 2,108), MP (8.82%, *n* = 2,510), RSV (7.38%, *n* = 1,799), and FluB (3.39%, *n* = 827). The majority of RTIs were caused by the single infection by one of the six detected respiratory pathogens (Table 1; Figure 2).

**Table 1:** Overall distribution of respiratory pathogens.

Respiratory pathogens	Single infection (%)	Mixed infection (%)	Total infection (%)
FluA	1791 (7.35)	317 (1.30)	2,108 (8.65)
FluB	618 (2.53)	209 (0.86)	827 (3.39)
ADV	1989 (8.16)	763 (3.13)	2,752 (11.29)
RSV	1,377 (5.65)	422 (1.73)	1799 (7.38)
HRV	2,208 (9.06)	1,028 (4.22)	3,236 (13.27)
MP	1817 (7.45)	693 (2.84)	2,510 (8.82)
Total	9,800 (40.20)	1,659 (6.80)	11,459 (47.00)

FluA, influenza virus A; FluB, influenza virus B; ADV, adenovirus; RSV, respiratory syncytial virus; HRV, human rhinovirus; MP, mycoplasma pneumonia.

Specifically, 1,546/1,659 (93.19%), 112/1,659 (6.75%), and 1/1,659 (0.06%) participants were infected with 2, 3, and 4 respiratory pathogens, respectively. The most frequent co-infections were caused by combinations of HRV + ADV (20.01%, 332/1,659), followed by HRV + MP (15.01%, 249/1,659), ADV + MP (12.78%, 212/1,659), HRV + RSV (10.85%, 180/1,659), FluA + HRV (7.96%, 132/1,659), FluA + RSV (3.38%, 56/1,659), FluB + MP (3.32%, 55/1,659), RSV + ADV (3.32%, 55/1,659), FluA + MP (3.25%, 54/1,659), FluA + ADV (3.13%, 52/1,659), FluB + RSV (2.77%, 46/1,659), FluB + HRV (2.65%, 44/1,659), RSV + MP (2.53%, 42/1,659), FluB + ADV (1.99%, 33/1,659), FluA + FluB (0.24%, 4/1,659), HRV + ADV + MP (2.59%, 43/1,659), HRV + ADV + RSV (0.72%, 12/1,659), HRV + MP + RSV (0.48%, 8/1,659), HRV + FluB + MP (0.42%, 7/1,659), MP + FluB + ADV (0.36%, 6/1,659), FluB + MP + RSV (0.30%, 5/1,659), MP + ADV + RSV (0.30%, 5/1,659), HRV + ADV + FluA (0.30%, 5/1,659), FluB + HRV + RSV (0.24%, 4/1,659), HRV + FluA + MP (0.24%, 4/1,659), FluA + RSV + ADV (0.18%, 3/1,659), HRV + FluB + ADV (0.18%, 3/1,659), HRV + FluA + RSV (0.18%, 3/1,659), FluA + RSV + MP (0.06%, 1/1,659), FluB + ADV + RSV (0.06%, 1/1,659), MP + FluA + ADV (0.06%, 1/1,659), ADV + FluA + FluB (0.06%, 1/1,659), and FluB + RSV + MP + HRV (0.06%, 1/1,659) (Table 2).

**Table 2:** Numbers of mixed infection cases with different combinations of respiratory pathogens.

	FluA	FluB	ADV	RSV	HRV	MP
Double pathogen positive (1,546 cases)						
FluA	-	4	52	56	132	54
FluB	4	-	33	46	44	55
ADV	52	33	-	55	332	212
RSV	56	46	55	-	180	42
HRV	132	44	332	180	-	249
MP	54	55	212	42	249	-

Combination name	Number	Combination name	Number
Triple pathogen positive (112 cases)			
HRV + ADV + MP	43	HRV + ADV + RSV	12
HRV + MP + RSV	8	HRV + FluB + MP	7
MP + FluB + ADV	6	FluB + MP + RSV	5
MP + ADV + RSV	5	HRV + ADV + FluA	5
FluB + HRV + RSV	4	HRV + FluA + MP	4
FluA + RSV + ADV	3	HRV + FluB + ADV	3
HRV + FluA + RSV	3	FluA + RSV + MP	1
FluB + ADV + RSV	1	MP + FluA + ADV	1
ADV + FluA + FluB	1		
Quadruple pathogen positive (1 case)			
FluB + RSV + MP + HRV	1		

FluA, influenza virus A; FluB, influenza virus B; ADV, adenovirus; RSV, respiratory syncytial virus; HRV, human rhinovirus; MP, mycoplasma pneumonia.

### 3.3 Sex-associated differences in detection rate of RTIs

**Figure 3:**
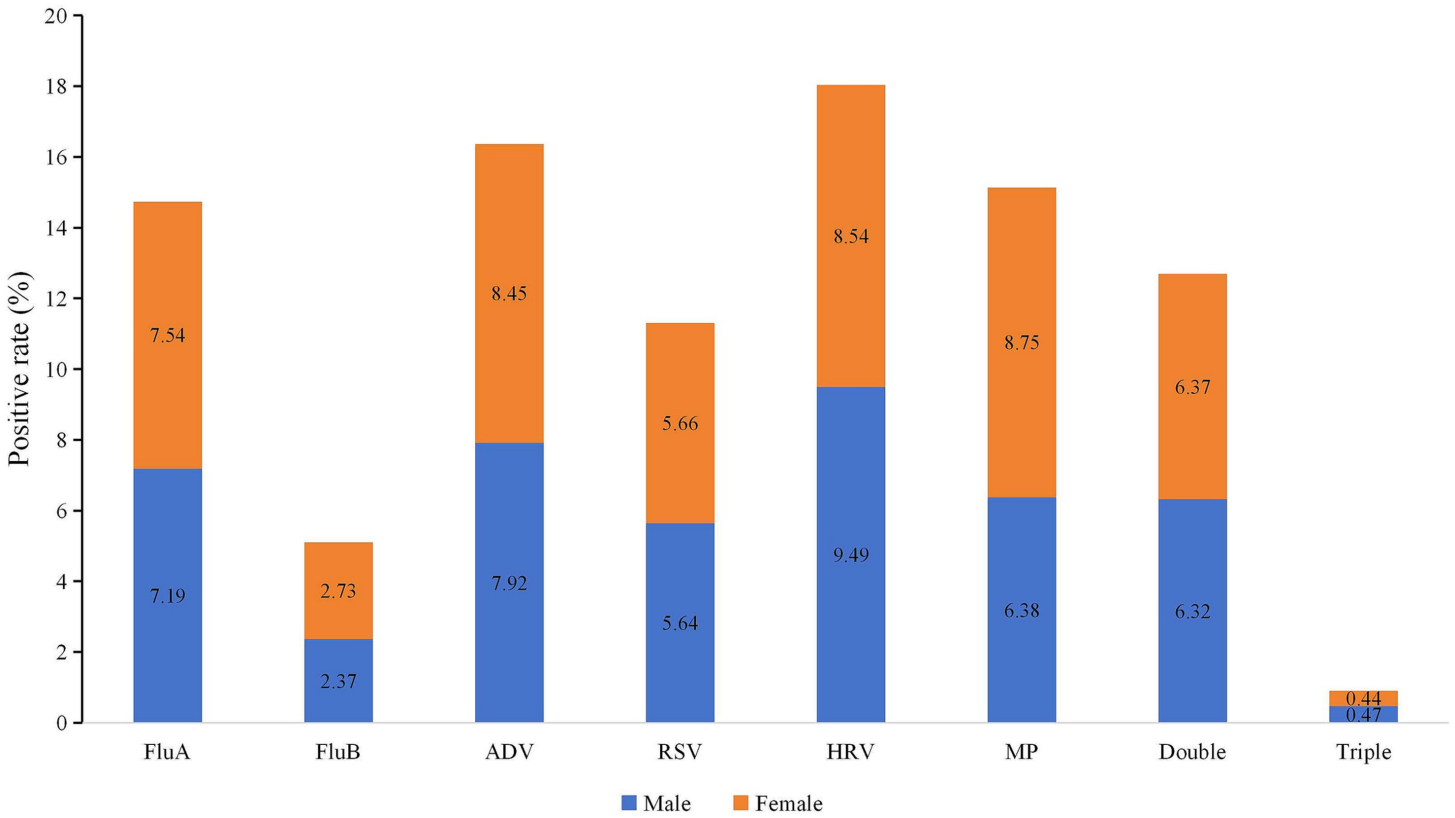
The positivity rate of respiratory pathogens in different sexes.

Sex preference was seen in RTIs during the two-year period in our medical institution, with females (48.47%, 5,364/11,066) more frequently affected than males (45.78%, 6,095/13,314) (χ^2^ = 17.606, *p* < 0.001, Table 3 and Figure 3). The positive rate of each respiratory pathogen in male and female participants is shown in Table 3. In male cases of RTIs caused by a single pathogen, HRV infection (9.49%, 1,263/13,314) was the most common, followed by ADV (7.92%, 1,054/13,314), FluA (7.19%, 957/13,314), MP (6.38%, 849/13,314), RSV (5.64%, 751/13,314), and FluB infections (2.37%, 316/13,314). MP infection (8.75%, 968/11,066) accounted for the largest propotion in female RTIs with a single respiratory pathogen, followed by HRV (8.54%, 945/11,066), ADV (8.45%, 935/11,066), FluA (7.54%, 834/11,066), RSV (5.66%, 626/11,066), and FluB infections (2.73%, 302/11,066). However, the positive rates of a single pathogen (FluA, FluB, ADV and RSV) and multiple pathogens were comparable between male and female participants (*p* > 0.05). Positivity for MP or HRV infection was significantly different between the two sex groups (*p* < 0.05).

**Table 3:** Analysis of respiratory pathogen detection rates by sex.

Sex	Total positive rate (%)	Double infection (%)	Triple infection (%)	Quadruple infection (%)	Single infection (%)					
					FluA	FluB	ADV	RSV	HRV	MP
Male (*n* = 13,314)	6,095 (45.78)	841 (6.32)	63 (0.47)	1	957 (7.19)	316 (2.37)	1,054 (7.92)	751 (5.64)	1,263 (9.49)	849 (6.38)
Female (*n* = 11,066)	5,364 (48.47)	705 (6.37)	49 (0.44)	0	834 (7.54)	302 (2.73)	935 (8.45)	626 (5.66)	945 (8.54)	968 (8.75)
χ^2^	17.606	0.030	0.122	-	1.079	3.094	2.290	0.003	6.574	49.245
*P*	<0.001	0.863	0.727	1.000	0.299	0.079	0.130	0.956	0.010	<0.001
95% CI	(−3.95, −1.43%)	(−0.36, 0.26%)	(−0.06, 0.12%)	(−0.01, 0.03%)	(−0.79, 0.09%)	(−0.74, 0.02%)	(−1.19, 0.13%)	(−0.57, 0.53%)	(0.21, 1.69%)	(−2.97, −1.77%)

FluA, influenza virus A; FluB, influenza virus B; ADV, adenovirus; RSV, respiratory syncytial virus; HRV, human rhinovirus; MP, mycoplasma pneumonia; CI, confidence interval.

Furthermore, the overall positive rate of RTIs in male participants differed significantly between the two time periods (2023–2024 vs. 2024–2025) (*p* < 0.001). Except for four-pathogen infections, the detection rate of co-infections caused by two or three respiratory pathogens was similar in male participants in 2023–2024 versus 2024–2025. Consistently in female participants, a significant difference in the detection rate of RTIs was seen between the two time periods (*p* < 0.001). The positivity for a single or multiple respiratory pathogens in female participants also differed significantly between the two time periods (Table 4).

**Table 4:** Changes in respiratory pathogen detection between different sexes from 2023 to 2025.

Respiratory pathogens	Male					Female				
	2023–2024 (*n* = 7,818)	2024–2025 (*n* = 5,496)	χ^2^	*P*	95% CI	2023–2024 (*n* = 6,627)	2024–2025 (*n* = 4,439)	χ^2^	*P*	95% CI
FluA (%)	327 (4.18)	630 (11.46)	256.399	<0.001	(−8.30, −6.27%)	292 (4.41)	542 (12.21)	232.312	<0.001	(−8.19, −6.86%)
FluB (%)	314 (4.02)	2 (0.04)	220.622	<0.001	(3.69, 4.27%)	300 (4.53)	2 (0.05)	201.156	<0.001	(3.98, 4.98%)
ADV (%)	956 (12.23)	98 (1.78)	482.996	<0.001	(9.91, 10.99%)	836 (12.62)	99 (2.23)	370.619	<0.001	(9.89, 11.10%)
RSV (%)	283 (3.62)	468 (8.52)	145.311	<0.001	(−5.33, −4.45%)	254 (3.83)	372 (8.38)	103.004	<0.001	(−4.90, −4.10%)
HRV (%)	672 (8.60)	591 (10.75)	17.499	<0.001	(−2.63, −1.67%)	460 (6.94)	485 (10.93)	54.039	<0.001	(−4.45, −3.53%)
MP (%)	771 (9.86)	78 (1.42)	385.308	<0.001	(7.94, 8.94%)	885 (13.35)	83 (1.87)	439.257	<0.001	(10.87, 12.09%)
Double infection (%)	607 (7.76)	234 (4.26)	67.055	<0.001	(3.07, 4.07%)	510 (7.70)	195 (4.39)	48.618	<0.001	(2.91, 3.71%)
Triple infection (%)	55 (0.70)	8 (0.15)	21.333	<0.001	(0.35, 0.75%)	41 (0.62)	8 (0.18)	11.593	<0.001	(0.26, 0.62%)
Quadruple infection (%)	1	0	-	1.000	(−0.01, 0.21%)	0	0	-	-	-
Total	3,986 (49.83)	2,109 (38.37)	206.793	<0.001	(9.93, 12.99%)	3,578 (53.99)	1786 (40.23)	201.429	<0.001	(11.83, 15.69%)

FluA, influenza virus A; FluB, influenza virus B; ADV, adenovirus; RSV, respiratory syncytial virus; HRV, human rhinovirus; MP, mycoplasma pneumonia; CI, confidence interval.

### 3.4 Detection rate of RTIs by age group

**Figure 4:**
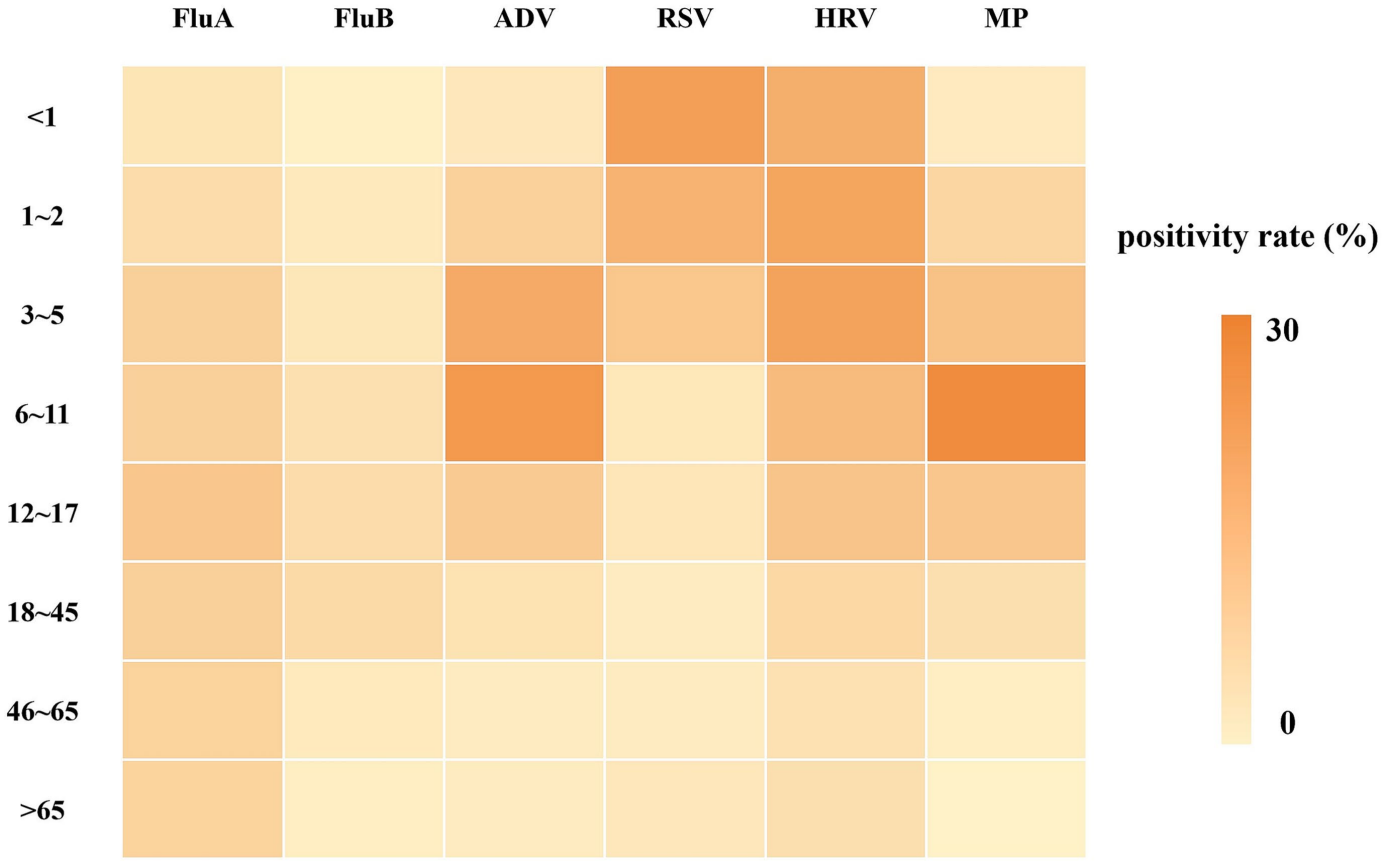
Heat map displays positivity rate of respiratory pathogens infections in different age groups, with each row representing an age group and each column representing a pathogen.

The positivity rates of RTIs caused by the six respiratory pathogens varied significantly across age groups (*p* < 0.001, Table 5 and Figure 4). RSV (22.35%) was the most frequently detected respiratory pathogen in infants, followed by HRV (18.09%). Meanwhile, patients between 1 ~ 2 years of age were mainly infected with HRV (20.91%) and RSV (17.21%). HRV infection (21.36%) was the most common cause of RTIs in preschool children, followed by ADV infection (19.30%). In children, MP (27.48%) and ADV (24.14%) were the most frequent causes of RTIs. Teenagers were the most vulnerable to HRV (12.72%) and FluA infections (11.97%). Similarly, FluA and HRV were the frequent respiratory pathogens leading to RTIs in adults. Positive rates of FluA, FluB, ADV, and MP were initially elevated and subsequently declined with age (*p* < 0.001). The positive rate of RSV peaked at 0–1 year, declined with age, and later rose in older adults. On the contrary, the positive rate of HRV increased first, then decreased, and increased again after 65 years of age.

**Table 5:** Analysis of respiratory pathogen detection rates by age.

Age (years)	FluA (%)	FluB (%)	ADV (%)	RSV (%)	HRV (%)	MP (%)
<1(*n* = 1,432)	57 (3.98)	14 (0.98)	47 (3.28)	320 (22.35)	259 (18.09)	35 (2.44)
1 ~ 2 (*n* = 2,702)	169 (6.25)	75 (2.78)	250 (9.25)	465 (17.21)	565 (20.91)	208 (7.70)
3 ~ 5 (*n* = 4,518)	421 (9.32)	166 (3.67)	872 (19.30)	549 (12.15)	965 (21.36)	608 (13.46)
6 ~ 11 (*n* = 5,190)	501 (9.65)	268 (5.16)	1,253 (24.14)	172 (3.31)	777 (14.97)	1,426 (27.48)
12 ~ 17 (*n* = 802)	96 (11.97)	49 (6.11)	88 (10.97)	28 (3.49)	102 (12.72)	94 (11.72)
18 ~ 45 (*n* = 1,449)	136 (9.39)	99 (6.83)	68 (4.69)	33 (2.28)	107 (7.38)	78 (5.38)
46 ~ 65 (*n* = 2,402)	214 (8.91)	63 (2.62)	50 (2.08)	52 (2.16)	139 (4.79)	34 (1.42)
>65 (*n* = 5,885)	514 (8.73)	93 (1.58)	124 (2.11)	180 (3.06)	322 (5.47)	27 (0.46)
χ^2^	80.767	98.697	2010.609	1456.667	907.310	2684.271
*P*	<0.001	<0.001	<0.001	<0.001	<0.001	<0.001

Infant, <1 year; toddler, 1 ~ 2 years; preschool children, 3 ~ 5 years; children, 6 ~ 11 years; teenagers, 12 ~ 17 years; young adult, 18 ~ 45 years; middle aged, 45 ~ 65 years; older adults, >65 years; FluA, influenza virus A; FluB, influenza virus B; ADV, adenovirus; RSV, respiratory syncytial virus; HRV, human rhinovirus; MP, mycoplasma pneumonia.

The positive rates of the six respiratory pathogens across all age groups in 2023–2024 and 2024–2025 are listed in Table 6. Significant differences in the positive rates of FluA, FluB, ADV, and MP across all age groups were detected between the two time periods (*p* < 0.05). Except for RSV infection, the positive rate of each respiratory pathogen in infants was significantly different in 2023–2024 versus 2024–2025 (*p* < 0.05). Except for the infants group, the positive rate of HRV showed no significant differences (*p* > 0.05) across age groups. Among young adults, positive rates of FluB, ADV, and MP were significantly different between the two time periods (*p* < 0.001).

**Table 6:** Changes in respiratory pathogen detection among different age groups from 2023 to 2025.

Respiratory pathogens		FluA (%)	FluB (%)	ADV (%)	RSV (%)	HRV (%)	MP (%)
<1	2023–2024 (*n* = 622)	17 (2.73)	14 (2.25)	33 (5.31)	130 (20.9)	98 (15.76)	30 (4.82)
	2024–2025 (*n* = 810)	40 (4.94)	0	14 (1.73)	190 (23.46)	161 (19.88)	5 (0.62)
	χ^2^	4.476	18.412	14.182	1.325	4.033	26.101
	*P*	0.034	<0.001	<0.001	0.250	0.045	<0.001
1–2	2023–2024 (*n* = 1,423)	60 (4.22)	75 (5.27)	190 (13.35)	185 (13.00)	284 (19.96)	189 (13.28)
	2024–2025 (*n* = 1,249)	109 (8.73)	0	60 (4.80)	280 (22.42)	281 (22.50)	19 (1.52)
	χ^2^	21.299	69.335	60.177	37.375	1.650	131.914
	*P*	<0.001	<0.001	<0.001	<0.001	0.199	<0.001
3–5	2023–2024 (*n* = 2,604)	153 (5.88)	162 (6.22)	761 (29.22)	107 (4.11)	564 (21.66)	559 (21.47)
	2024–2025 (*n* = 1914)	268 (14.00)	4 (0.21)	111 (5.80)	442 (23.09)	401 (20.95)	49 (2.56)
	χ^2^	86.216	112.668	388.643	372.433	0.329	338.602
	*P*	<0.001	<0.001	<0.001	<0.001	0.566	<0.001
6–11	2023–2024 (*n* = 3,691)	119 (3.22)	268 (7.26)	1,158 (31.37)	107 (2.90)	491 (13.30)	1,316 (35.65)
	2024–2025 (*n* = 1,499)	382 (25.48)	0	95 (6.34)	65 (4.34)	286 (19.08)	110 (7.34)
	χ^2^	605.661	114.767	364.862	6.873	27.946	428.953
	*P*	<0.001	<0.001	<0.001	0.009	0.566	<0.001
12–17	2023–2024 (*n* = 460)	21 (4.57)	48 (10.43)	77 (16.74)	22 (4.78)	53 (11.52)	85 (18.48)
	2024–2025 (*n* = 342)	75 (21.93)	1 (0.29)	11 (3.22)	6 (1.75)	49 (14.33)	9 (2.63)
	χ^2^	56.132	35.176	36.720	5.339	1.391	47.607
	*P*	<0.001	<0.001	<0.001	0.021	0.238	<0.001
18–45	2023–2024 (*n* = 920)	76 (8.26)	99 (10.76)	57 (6.20)	25 (2.72)	61 (6.63)	72 (7.83)
	2024–2025 (*n* = 529)	60 (11.34)	0	11 (2.08)	8 (1.51)	46 (8.70)	6 (1.13)
	χ^2^	3.749	61.099	12.724	2.192	2.095	29.531
	*P*	0.053	<0.001	<0.001	0.139	0.148	<0.001
46–65	2023–2024 (*n* = 1,367)	81 (5.93)	63 (4.61)	44 (3.22)	40 (2.93)	86 (6.29)	29 (2.12)
	2024–2025 (*n* = 1,035)	133 (12.85)	0	6 (0.58)	12 (1.16)	53 (5.12)	5 (0.48)
	χ^2^	34.805	48.984	20.126	8.680	1.480	11.330
	*P*	<0.001	<0.001	<0.001	0.003	0.224	0.001
>65	2023–2024 (*n* = 3,358)	213 (6.34)	93 (2.77)	114 (3.39)	132 (3.93)	193 (5.75)	22 (0.66)
	2024–2025 (*n* = 2,527)	301 (11.91)	0	10 (0.40)	48 (1.90)	129 (5.10)	5 (0.20)
	χ^2^	56.087	71.109	62.880	20.068	1.151	6.602
	*P*	<0.001	<0.001	<0.001	<0.001	0.283	0.010

Infant, <1 year; toddler, 1 ~ 2 years; preschool children, 3 ~ 5 years; children, 6 ~ 11 years; teenagers, 12 ~ 17 years; young adult, 18 ~ 45 years; middle aged, 45 ~ 65 years; older adults, >65 years; FluA, influenza virus A; FluB, influenza virus B; ADV, adenovirus; RSV, respiratory syncytial virus; HRV, human rhinovirus; MP, mycoplasma pneumonia.

### 3.5 Detection rates of RTIs in seasons

**Figure 5:**
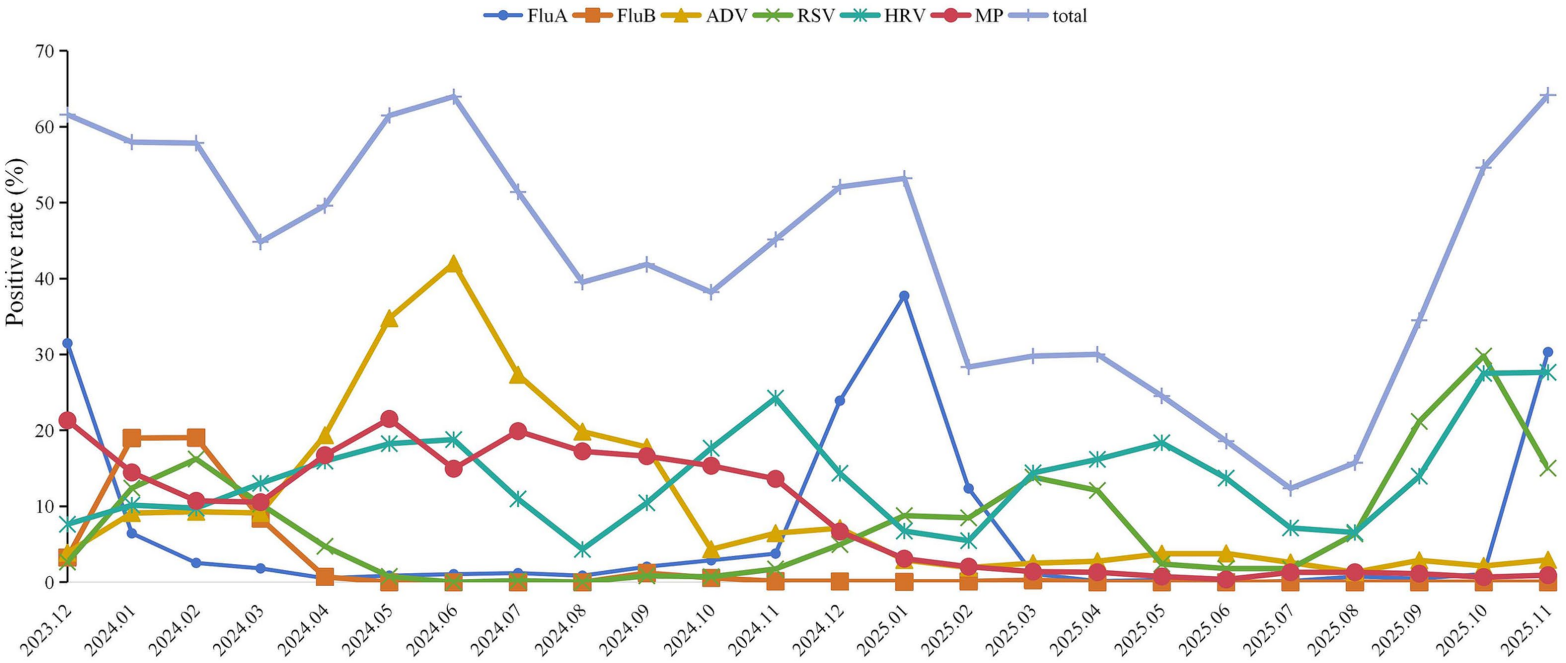
Positivity rates of respiratory pathogens in each month.

The overall detection rate of RTIs peaked in November 2025 (64.15%), and hit the bottom in July 2025 (12.35%). The seasonal prevalence of RTIs in our medical institution fluctuated, showing an overall downward trend from December 2023 to March 2024, an upward trend from April 2024 to June 2024, a downward trend again from July 2024 to July 2025, and finally an upward trend from August 2025 to November 2025. Classified by the monthly positive rate of respiratory pathogens, the highest positive rate of FluA was found in December 2023 (31.48%), the interval from December 2024 to February 2025, and November 2025. FluB infection was the most popular in January 2024 (18.99%) and February 2024 (19.05%). The prevalence of HRV infection peaked in March 2024 (13.02%), the interval from October 2024 to November 2024, and that from March 2025 to August 2025. ADV infection was popular in April (19.36%), May (34.77%), June (41.99%), July (27.33%), August (19.84%), and September 2024 (17.80%). RSV infection peaked in September (21.18%) and October 2025 (29.79%). FluA infection showed the lowest prevalence in January (6.44%), February (2.54%), March (1.82%), and April 2024 (0.52%). FluB was barely detected in May and August 2024, as well as the interval from October 2024 to November 2025. The lowest positive rate of RSV was seen in December 2023 (2.66%) and September 2024 (0.84%) (Table 7; Figure 5).

**Table 7:** Analysis of respiratory pathogen detection rates by month.

Time	Total positive rate (%)	Total positive case (*n*)	Total cases (*n*)	Respiratory pathogen (%)					
				FluA	FluB	ADV	RSV	HRV	MP
2023.12	61.59	855	1,388	437 (31.48)	45 (3.24)	54 (3.89)	37 (2.66)	106 (7.63)	296 (21.32)
2024.01	57.97	1,062	1832	118 (6.44)	348 (18.99)	167 (9.11)	227 (12.39)	186 (10.15)	265 (14.46)
2024.02	57.84	841	1,454	37 (2.54)	277 (19.05)	135 (9.28)	236 (16.23)	142 (9.76)	156 (10.72)
2024.03	44.82	688	1,535	28 (1.82)	129 (8.40)	140 (9.12)	158 (10.29)	200 (13.02)	162 (10.55)
2024.04	49.61	566	1,141	6 (0.52)	8 (0.70)	221 (19.36)	54 (4.73)	182 (15.95)	191 (16.73)
2024.05	61.47	640	1,041	9 (0.86)	0	362 (34.77)	8 (0.76)	190 (18.25)	224 (21.51)
2024.06	63.97	783	1,224	13 (1.06)	0	514 (41.99)	0	230 (18.79)	183 (14.95)
2024.07	51.42	726	1,412	17 (1.20)	0	386 (27.33)	3 (0.21)	155 (10.97)	281 (19.90)
2024.08	39.51	456	1,154	10 (0.86)	0	229 (19.84)	0	50 (4.33)	199 (17.24)
2024.09	41.87	348	831	17 (2.04)	10 (1.20)	148 (17.80)	7 (0.84)	87 (10.46)	138 (16.60)
2024.10	38.20	264	691	20 (2.89)	4 (0.57)	30 (4.34)	5 (0.72)	122 (17.65)	106 (15.34)
2024.11	45.14	335	742	28 (3.77)	1 (0.13)	48 (6.46)	13 (1.75)	180 (24.25)	101 (13.61)
2024.12	52.07	577	1,108	265 (23.91)	1 (0.09)	79 (7.12)	55 (4.96)	159 (14.35)	74 (6.67)
2025.01	53.19	891	1,675	632 (37.73)	1 (0.05)	49 (2.92)	147 (8.77)	113 (6.74)	52 (3.10)
2025.02	28.35	264	931	115 (12.35)	1 (0.10)	18 (1.93)	79 (8.48)	51 (5.47)	19 (2.04)
2025.03	29.79	213	715	8 (1.11)	2 (0.27)	18 (2.51)	99 (13.84)	103 (14.40)	10 (1.39)
2025.04	30.02	206	686	1 (0.14)	0	19 (2.76)	83 (12.09)	111 (16.18)	9 (1.31)
2025.05	24.54	163	664	2 (0.30)	0	25 (3.76)	16 (2.40)	122 (18.37)	5 (0.75)
2025.06	18.59	103	554	0	0	21 (3.79)	10 (1.80)	76 (13.71)	2 (0.36)
2025.07	12.35	76	615	1 (0.16)	0	16 (2.60)	11 (1.78)	44 (7.15)	8 (1.30)
2025.08	15.73	84	534	4 (0.74)	0	7 (1.31)	34 (6.36)	35 (6.55)	7 (1.31)
2025.09	34.51	215	623	3 (0.48)	0	18 (2.88)	132 (21.18)	87 (13.96)	7 (1.12)
2025.10	54.63	407	745	8 (1.07)	0	16 (2.14)	222 (29.79)	205 (27.51)	5 (0.67)
2025.11	64.15	696	1,085	329 (30.32)	0	32 (2.95)	163 (15.02)	300 (27.65)	10 (0.92)
Total	47.00	11,459	24,380	2,108 (8.65)	827 (3.39)	2,752 (11.29)	1799 (7.38)	3,236 (13.27)	2,510 (8.82)

FluA, influenza virus A; FluB, influenza virus B; ADV, adenovirus; RSV, respiratory syncytial virus; HRV, human rhinovirus; MP, mycoplasma pneumonia.

In our hospital located in Changzhou with a humid subtropical climate, RTIs in 2023–2024 showed a distinct seasonal trend peaking in winter (59.01%, 2,758/4,674), followed by summer (51.85%, 1,965/3,790), spring (50.96%, 1,894/3,717), and autumn (41.83%, 947/2,264). In 2024–2025, autumn (53.73%, 1,318/2,453) was the season when RTIs peaked, followed by winter (46.63%, 1,732/3,714), spring (28.18%, 582/2,065), and summer (15.44%, 263/1,703). Seasonal distribution of positivity for respiratory pathogens, either alone or in combination, showed significant differences across the four seasons from 2023 to 2025 (*p* < 0.05). However, FluB could barely be detected throughout the year in 2024–2025. FluA infection was popular in winter and autumn, while ADV was usually detected in summer. Positivity for HRV was frequently found in autumn and spring. RSV infection showed a seasonal trend in winter in 2023–2024 and autumn in 2024–2025. MP infection peaked in summer during 2023–2024 and winter during 2024–2025. Notably, the positive rate of MP across the four seasons was significantly higher in 2023–2024 than that in 2024–2025 (Table 8).

**Table 8:** Seasonal changes in respiratory pathogen detection from 2023 to 2025.

Season	Total positive rate (%)	Double infection (%)	Triple infection (%)	Quadruple infection (%)	Single infection (%)					
					FluA	FluB	ADV	RSV	HRV	MP
2023–2024										
Winter (*n* = 4,674)	2,758 (59.01)	428 (9.16)	40 (0.86)	1 (0.02)	498 (10.65)	496 (10.61)	214 (4.58)	347 (7.42)	244 (5.22)	490 (10.48)
Spring (*n* = 3,717)	1894 (50.96)	310 (8.34)	34 (0.91)	0	35 (0.94)	108 (2.91)	520 (13.99)	168 (4.52)	335 (9.01)	384 (10.33)
Summer (*n* = 3,790)	1965 (51.85)	271 (7.15)	17 (0.45)	0	33 (0.87)	0	895 (23.61)	2 (0.05)	238 (6.28)	509 (13.43)
Autumn (*n* = 2,264)	947 (41.83)	108 (4.77)	5 (0.22)	0	53 (2.34)	10 (0.44)	163 (7.20)	20 (0.88)	315 (13.91)	273 (12.06)
χ^2^	186.802	44.860	15.543	-	692.231	730.196	766.774	379.096	179.889	24.350
*P*	< 0.001	< 0.001	0.001	-	< 0.001	< 0.001	< 0.001	< 0.001	< 0.001	< 0.001
2024–2025										
Winter (*n* = 3,714)	1732 (46.63)	166 (4.47)	6 (0.16)	0	899 (24.21)	2 (0.05)	96 (2.58)	224 (6.03)	226 (6.09)	113 (3.04)
Spring (*n* = 2065)	582 (28.18)	49 (2.37)	1	0	10 (0.48)	2 (0.10)	38 (1.84)	169 (8.18)	292 (14.14)	21 (1.02)
Summer (*n* = 1703)	263 (15.44)	13 (0.76)	0	0	5 (0.47)	0	36 (2.11)	52 (3.05)	142 (8.34)	15 (0.88)
Autumn (*n* = 2,453)	1,318 (53.73)	201 (8.19)	9 (0.35)	0	258 (10.52)	0	27 (1.10)	395 (16.10)	416 (16.96)	12 (0.49)
χ^2^	811.797	160.397	9.449	-	1024.031	2.682	17.113	277.941	216.375	77.364
*P*	< 0.001	< 0.001	0.016	-	< 0.001	0.400	0.001	< 0.001	< 0.001	< 0.001

FluA, influenza virus A; FluB, influenza virus B; ADV, adenovirus; RSV, respiratory syncytial virus; HRV, human rhinovirus; MP, mycoplasma pneumonia.

### 3.6 Differences in the detection rate between inpatients and outpatients

**Figure 6:**
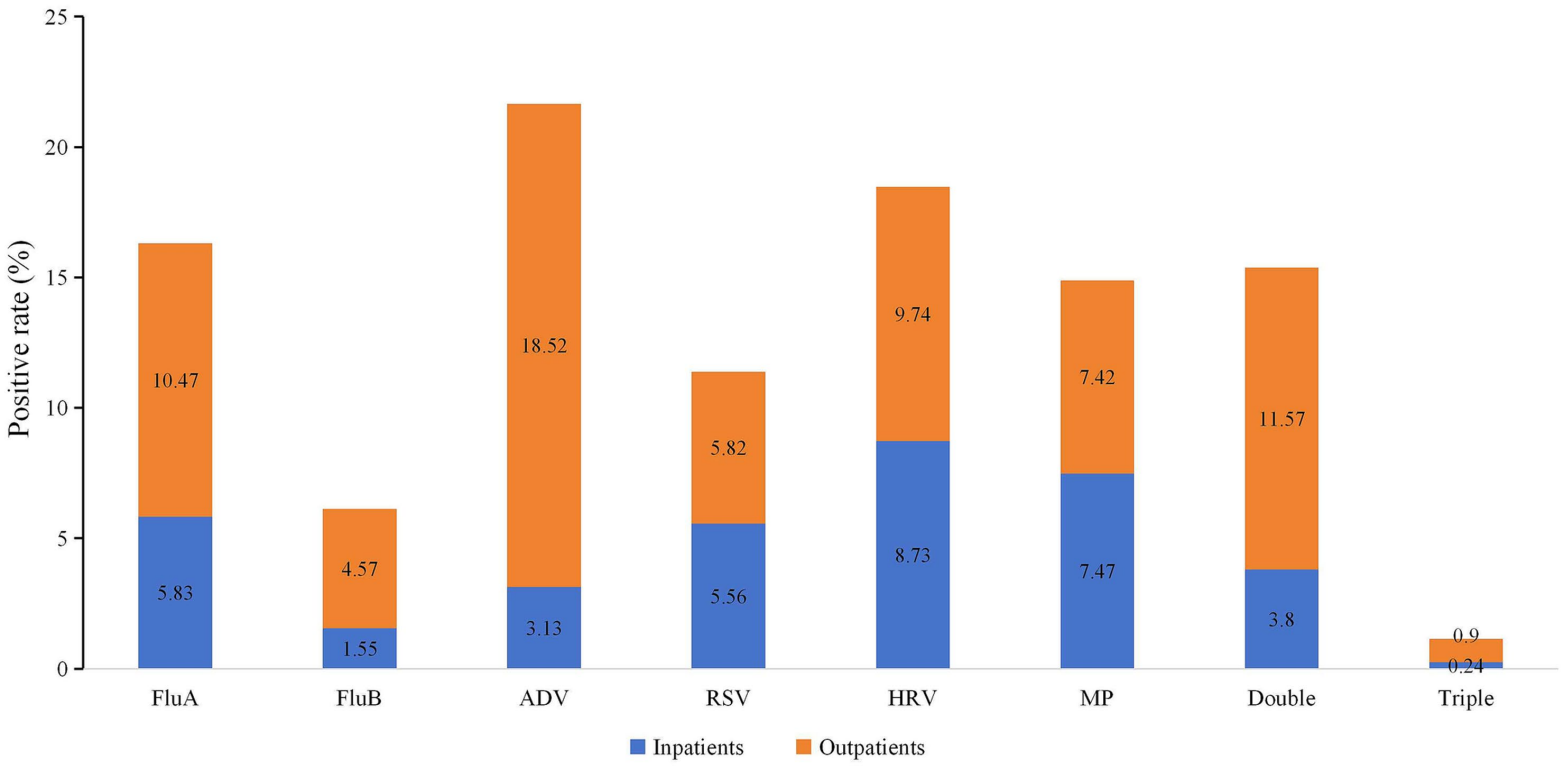
The positivity rate of respiratory pathogens in different patients.

There are statistically significant differences in age and sex between inpatients and outpatients (Table 9). The overall detection rate of RTIs differed significantly between inpatients and outpatients from December 2023 to November 2025 (χ^2^ = 2302.299, *p* < 0.001). Details of the positivity for each respiratory pathogen in both inpatients and outpatients are shown in Table 10 and Figure 6. RTIs in inpatients were mostly caused by HRV (8.73%, 1,432/16,412), followed by MP (7.47%, 1,226/16,412), FluA (5.83%, 957/16,412), RSV (5.56%, 913/16,412), ADV (3.13%, 513/16,412) and FluB (1.55%, 254/16,412). ADV (18.52%, 1476/7968) was the major driver of RTIs in outpatients, followed by FluA (10.47%, 834/7,968), HRV (9.74%, 776/7,968), MP (7.42%, 591/7,968), RSV (5.82%, 464/7,968), and FluB (4.57%, 364/7,968). The positive rates of single infections caused by FluA, FluB, ADV, and HRV, as well as co-infections caused by two and three pathogens were significantly different between inpatients and outpatients (*p* < 0.05). Positivity for MP and RSV, and co-infections caused by four respiratory pathogens was comparable between groups (*p* > 0.05).

**Table 9:** Comparison of clinical parameters between inpatients and outpatients.

Variables	Total (*n* = 24,380)	Inpatients (*n* = 16,412)	Outpatients (*n* = 7,968)	Statistic	*P*
Age, M (Q₁, Q₃)	8.00 (4.00, 64.00)	41.00 (4.00, 72.00)	6.00 (3.00, 9.00)	Z = −96.61	<0.001
Sex, *n* (%)				χ^2^ = 74.80	<0.001
Female	11,066 (45.39)	7,134 (43.47)	3,932 (49.35)		
Male	13,314 (54.61)	9,278 (56.53)	4,036 (50.65)		

Z: Mann–Whitney test, χ^2^: Chi-square test, M: Median, Q₁: 1st Quartile, Q₃: 3st Quartile.

**Table 10:** Analysis of respiratory pathogen detection rates by case type.

Case type	Total positive rate (%)	Double infection (%)	Triple infection (%)	Quadruple infection (%)	Single infection (%)					
					FluA	FluB	ADV	RSV	HRV	MP
Inpatients (*n* = 16,412)	5,960 (36.31)	624 (3.80)	40 (0.24)	1	957 (5.83)	254 (1.55)	513 (3.13)	913 (5.56)	1,432 (8.73)	1,226 (7.47)
Outpatients (*n* = 7,968)	5,499 (69.01)	922 (11.57)	72 (0.90)	0	834 (10.47)	364 (4.57)	1,476 (18.52)	464 (5.82)	776 (9.74)	591 (7.42)
χ^2^	2302.299	545.135	51.078	-	169.354	198.092	1697.398	0.682	6.691	0.022
*P*	<0.001	<0.001	<0.001	1.000	<0.001	<0.001	<0.001	0.409	0.010	0.883

FluA, influenza virus A; FluB, influenza virus B; ADV, adenovirus; RSV, respiratory syncytial virus; HRV, human rhinovirus; MP, mycoplasma pneumonia.

In both inpatients and outpatients, the overall detection rate of RTIs differed significantly in 2023–2024 versus 2024–2025 (*p* < 0.001). Except for co-infections caused by four pathogens, positivity for one, two, or three pathogens differed significantly in inpatients between the two time periods. In outpatients, a significant difference was detected in the positive rate of single infections and co-infections in 2023–2024 versus 2024–2025 (*p* < 0.001) (Table 11).

**Table 11:** Changes in respiratory pathogen detection between different patients from 2023 to 2025.

Type	Inpatients				Outpatients			
	2023–2024 (*n* = 8,998)	2024–2025 (*n* = 7,414)	χ^2^	*P*	2023–2024 (*n* = 5,447)	2024–2025 (*n* = 2,521)	χ^2^	*P*
FluA (%)	375 (4.17)	582 (7.85)	100.380	<0.001	244 (4.48)	590 (23.40)	658.568	<0.001
FluB (%)	253 (2.81)	1 (0.01)	208.887	<0.001	361 (6.63)	3 (0.12)	167.455	<0.001
ADV (%)	408 (4.53)	105 (1.42)	130.513	<0.001	1,384 (25.41)	92 (3.65)	540.627	<0.001
RSV (%)	330 (3.67)	583 (7.86)	136.226	<0.001	207 (3.80)	257 (8.80)	128.478	<0.001
HRV (%)	651 (7.23)	781 (10.53)	55.554	<0.001	481 (8.83)	295 (11.70)	16.162	<0.001
MP (%)	1,103 (12.26)	123 (1.66)	660.658	<0.001	553 (10.15)	38 (1.51)	187.563	<0.001
Double infection (%)	443 (4.92)	181 (2.44)	68.462	<0.001	674 (12.37)	248 (9.84)	10.835	0.001
Triple infection (%)	36 (0.40)	4 (0.05)	20.031	<0.001	60 (1.10)	12 (0.48)	7.531	0.006
Quadruple infection (%)	1	0	-	1.000	0	0	-	-
Total	3,600 (40.01)	2,360 (31.83)	117.524	<0.001	3,964 (72.77)	1,535 (60.89)	113.843	<0.001

### 3.7 Independent risk factors of RTIs

The variables associated with six common respiratory pathogens infections were analyzed by a multivariate logistic regression analysis. We found that age, inpatients, autumn and winter were independent factors of FluA infection (*p* < 0.001, OR = 1.01 (95% CI 1.01, 1.01); *p* < 0.001, OR = 0.34 (95% CI 0.31, 0.38); *p* < 0.001, OR = 10.23 (95% CI 7.68, 13.64); *p* < 0.001, OR = 24.17 (95% CI 18.38, 31.79)) (Table 12) and FluB infection (*p* = 0.003, OR = 0.99 (95% CI 0.99, 0.99); *p* < 0.001, OR = 0.36 (95% CI 0.30, 0.42); *p* < 0.001, OR = 0.12 (95% CI 0.07, 0.21); *p* < 0.001, OR = 3.34 (95% CI 2.77, 4.03)) (Table 13). Age, inpatient, summer, autumn and winter were significant predictors of ADV infection (*p* < 0.001, OR = 0.98 (95% CI 0.98, 0.98); *p* < 0.001, OR = 0.19 (95% CI 0.17, 0.21); *p* < 0.001, OR = 1.48 (95% CI 1.33, 1.65); *p* < 0.001, OR = 0.33 (95% CI 0.28, 0.38); *p* < 0.001, OR = 0.32 (95% CI 0.28, 0.36)) (Table 14) and RSV infection (*p* < 0.001, OR = 0.97 (95% CI 0.97, 0.98); *p* < 0.001, OR = 1.41 (95% CI 1.27, 1.57); *p* < 0.001, OR = 0.13 (95% CI 0.10, 0.17); *p* < 0.001, OR = 1.49 (95% CI 1.30, 1.71); *p* < 0.001, OR = 1.39 (95% CI 1.23, 1.58)) (Table 15). However, age, female, inpatients, summer and winter might be protective factors (*p* < 0.001, OR = 0.98 (95% CI 0.98, 0.98); *p* < 0.001, OR = 0.86 (95% CI 0.80, 0.93); *p* < 0.001, OR = 0.87 (95% CI 0.80, 0.94); *p* < 0.001, OR = 0.60 (95% CI 0.53, 0.67); *p* < 0.001, OR = 0.52 (95% CI 0.47, 0.58)) and autumn might be a risk factor (*p* < 0.001, OR = 1.27 (95% CI 1.15, 1.41)) for HRV infection (Table 16). In addition, age, female, inpatient, summer and autumn were significant predictors for MP infection (*p* < 0.001, OR = 0.96 (95% CI 0.96, 0.96); *p* < 0.001, OR = 1.25 (95% CI 1.15, 1.37); *p* < 0.001, OR = 1.51 (95% CI 1.38, 1.65); *p* = 0.003, OR = 1.20 (95% CI 1.06, 1.35); *p* < 0.001, OR = 0.61 (95% CI 0.53, 0.70)) (Table 17). These results suggest that the six types of infections might arise from various independent risk factors.

**Table 12:** Results of multivariate logistic regression analysis on the impact of FluA infection.

Variable	Logistic regression					Multivariate logistic regression				
	β	S.E	Z	*P*	OR (95% CI)	β	S.E	Z	*P*	OR (95% CI)
Age	0.00	0.00	1.35	0.178	1.00 (1.00 ~ 1.00)	0.01	0.00	9.15	<0.001	1.01 (1.01 ~ 1.01)
Sex										
Male					1.00 (Reference)					1.00 (Reference)
Female	0.06	0.05	1.24	0.213	1.06 (0.97 ~ 1.16)	−0.01	0.05	−0.23	0.820	0.99 (0.90 ~ 1.09)
Case type										
Outpatients					1.00 (Reference)					1.00 (Reference)
Inpatients	−0.77	0.05	−16.70	<0.001	0.46 (0.42 ~ 0.51)	−1.07	0.06	−18.29	<0.001	0.34 (0.31 ~ 0.38)
Season										
Spring					1.00 (Reference)					1.00 (Reference)
Summer	−0.13	0.20	−0.65	0.514	0.88 (0.59 ~ 1.30)	−0.21	0.20	−1.01	0.312	0.81 (0.55 ~ 1.21)
Autumn	2.30	0.15	15.72	<0.001	9.96 (7.48 ~ 13.27)	2.33	0.15	15.86	<0.001	10.23 (7.68 ~ 13.64)
Winter	3.22	0.14	23.10	<0.001	25.08 (19.08 ~ 32.97)	3.19	0.14	22.80	<0.001	24.17 (18.38 ~ 31.79)

OR, Odds Ratio; CI, Confidence Interval.

**Table 13:** Results of multivariate logistic regression analysis on the impact of FluB infection.

Variable	Logistic regression					Multivariate logistic regression				
	β	S. E	Z	*P*	OR (95% CI)	*β*	S. E	Z	*P*	OR (95% CI)
Age	−0.01	0.00	−7.58	<0.001	0.99 (0.99 ~ 0.99)	−0.01	0.00	−3.02	0.003	0.99 (0.99 ~ 0.99)
Sex										
Male					1.00 (Reference)					1.00 (Reference)
Female	0.14	0.07	2.03	0.042	1.15 (1.01 ~ 1.33)	0.02	0.07	0.31	0.754	1.02 (0.89 ~ 1.18)
Case type										
Outpatients					1.00 (Reference)					1.00 (Reference)
Inpatients	−1.12	0.07	−15.60	<0.001	0.32 (0.28 ~ 0.37)	−1.02	0.08	−12.19	<0.001	0.36 (0.30 ~ 0.42)
Season										
Spring					1.00 (Reference)					1.00 (Reference)
Summer	−15.86	145.10	−0.11	0.913	0.00 (0.00 ~ ∞)	−16.95	234.19	−0.07	0.942	0.00 (0.00 ~ ∞)
Autumn	−2.04	0.27	−7.50	<0.001	0.13 (0.08 ~ 0.22)	−2.12	0.27	−7.76	<0.001	0.12 (0.07 ~ 0.21)
Winter	1.26	0.09	13.34	<0.001	3.54 (2.94 ~ 4.26)	1.21	0.10	12.64	<0.001	3.34 (2.77 ~ 4.03)

OR, Odds Ratio; CI, Confidence Interval.

**Table 14:** Results of multivariate logistic regression analysis on the impact of ADV infection.

Variable	Logistic regression					Multivariate logistic regression				
	β	S. E	Z	*P*	OR (95% CI)	β	S. E	Z	*P*	OR (95% CI)
Age	−0.03	0.00	−28.70	<0.001	0.97 (0.97 ~ 0.97)	−0.02	0.00	−16.66	<0.001	0.98 (0.98 ~ 0.98)
Sex										
Male					1.00 (Reference)					1.00 (Reference)
Female	0.04	0.04	1.09	0.275	1.05 (0.97 ~ 1.13)	−0.06	0.04	−1.27	0.203	0.94 (0.87 ~ 1.03)
Case type										
Outpatients					1.00 (Reference)					1.00 (Reference)
Inpatients	−2.00	0.05	−43.58	<0.001	0.14 (0.12 ~ 0.15)	−1.66	0.05	−32.87	<0.001	0.19 (0.17 ~ 0.21)
Season										
Spring					1.00 (Reference)					1.00 (Reference)
Summer	0.55	0.05	10.82	<0.001	1.73 (1.57 ~ 1.91)	0.39	0.06	7.09	<0.001	1.48 (1.33 ~ 1.65)
Autumn	−0.87	0.07	−12.12	<0.001	0.42 (0.37 ~ 0.48)	−1.12	0.08	−14.92	<0.001	0.33 (0.28 ~ 0.38)
Winter	−0.90	0.06	−15.07	<0.001	0.41 (0.36 ~ 0.46)	−1.14	0.06	−17.90	<0.001	0.32 (0.28 ~ 0.36)

OR, Odds Ratio; CI, Confidence Interval.

**Table 15:** Results of multivariate logistic regression analysis on the impact of RSV infection.

Variable	Logistic regression					Multivariate logistic regression				
	β	S.E	Z	*P*	OR (95% CI)	β	S.E	Z	*P*	OR (95% CI)
Age	−0.02	0.00	−21.16	<0.001	0.98 (0.97 ~ 0.98)	−0.03	0.00	−22.51	<0.001	0.97 (0.97 ~ 0.98)
Sex										
Male					1.00 (Reference)					1.00 (Reference)
Female	−0.01	0.05	−0.18	0.861	0.99 (0.90 ~ 1.09)	−0.06	0.05	−1.26	0.209	0.94 (0.85 ~ 1.04)
Case type										
Outpatients					1.00 (Reference)					1.00 (Reference)
Inpatients	−0.13	0.05	−2.46	0.014	0.88 (0.80 ~ 0.97)	0.34	0.05	6.35	<0.001	1.41 (1.27 ~ 1.57)
Season										
Spring					1.00 (Reference)					1.00 (Reference)
Summer	−1.99	0.14	−14.06	<0.001	0.14 (0.10 ~ 0.18)	−2.03	0.14	−14.30	<0.001	0.13 (0.10 ~ 0.17)
Autumn	0.51	0.07	7.47	<0.001	1.67 (1.46 ~ 1.90)	0.40	0.07	5.78	<0.001	1.49 (1.30 ~ 1.71)
Winter	0.28	0.06	4.36	<0.001	1.32 (1.16 ~ 1.49)	0.33	0.06	5.16	<0.001	1.39 (1.23 ~ 1.58)

OR, Odds Ratio; CI, Confidence Interval.

**Table 16:** Results of multivariate logistic regression analysis on the impact of HRV infection.

Variable	Logistic regression					Multivariate logistic regression				
	β	S.E	Z	*P*	OR (95% CI)	β	S.E	Z	*P*	OR (95% CI)
Age	−0.02	0.00	−26.08	<0.001	0.98 (0.98 ~ 0.98)	−0.02	0.00	−23.22	<0.001	0.98 (0.98 ~ 0.98)
Sex										
Male					1.00 (Reference)					1.00 (Reference)
Female	−0.09	0.04	−2.49	0.013	0.91 (0.84 ~ 0.98)	−0.15	0.04	−3.83	<0.001	0.86 (0.80 ~ 0.93)
Case type										
Outpatients					1.00 (Reference)					1.00 (Reference)
Inpatients	−0.51	0.04	−13.20	<0.001	0.60 (0.56 ~ 0.65)	−0.14	0.04	−3.35	<0.001	0.87 (0.80 ~ 0.94)
Season										
Spring					1.00 (Reference)					1.00 (Reference)
Summer	−0.44	0.06	−7.72	<0.001	0.65 (0.58 ~ 0.72)	−0.52	0.06	−8.96	<0.001	0.60 (0.53 ~ 0.67)
Autumn	0.34	0.05	6.74	<0.001	1.41 (1.28 ~ 1.56)	0.24	0.05	4.64	<0.001	1.27 (1.15 ~ 1.41)
Winter	−0.63	0.05	−12.00	<0.001	0.53 (0.48 ~ 0.59)	−0.65	0.05	−12.12	<0.001	0.52 (0.47 ~ 0.58)

OR, Odds Ratio; CI, Confidence Interval.

**Table 17:** Results of multivariate logistic regression analysis on the impact of MP infection.

Variable	Logistic regression					Multivariate logistic regression				
	β	S.E	Z	*P*	OR (95% CI)	β	S.E	Z	*P*	OR (95% CI)
Age	−0.04	0.00	−28.26	<0.001	0.96 (0.96 ~ 0.96)	−0.04	0.00	−29.87	<0.001	0.96 (0.96 ~ 0.96)
Sex										
Male					1.00 (Reference)					1.00 (Reference)
Female	0.27	0.04	6.37	<0.001	1.31 (1.20 ~ 1.42)	0.23	0.04	5.20	<0.001	1.25 (1.15 ~ 1.37)
Case type										
Outpatients					1.00 (Reference)					1.00 (Reference)
Inpatients	−0.27	0.04	−6.17	<0.001	0.76 (0.70 ~ 0.83)	0.41	0.05	9.00	<0.001	1.51 (1.38 ~ 1.65)
Season										
Spring					1.00 (Reference)					1.00 (Reference)
Summer	0.20	0.06	3.32	<0.001	1.22 (1.08 ~ 1.37)	0.18	0.06	2.94	0.003	1.20 (1.06 ~ 1.35)
Autumn	−0.32	0.07	−4.59	<0.001	0.73 (0.63 ~ 0.83)	−0.49	0.07	−6.93	<0.001	0.61 (0.53 ~ 0.70)
Winter	−0.01	0.06	−0.23	0.821	0.99 (0.88 ~ 1.10)	0.05	0.06	0.86	0.390	1.05 (0.94 ~ 1.18)

OR, Odds Ratio; CI, Confidence Interval.

## 4 Discussion

RTIs can be affected by various factors, including pathogen type, sex, testing method, seasonal changes, geographical factors, population characteristics, and public health interventions (4, 9, 10). In this single-center, two-year, retrospective study, we analyzed the prevalence of RTIs by RT-PCR testing for six common respiratory pathogens across all age groups in a local hospital in Changzhou. Stratified by sex, age, season, and patient status, our data provided useful information to guide local management of RTIs and reduce antibiotic abuse.

The overall positivity for six common respiratory pathogens in Changzhou from December 2023 to November 2025, mainly involving single infections, was 47.00%, which was similar to that in Zhuhai (Guangdong, China) (44.48%) (11). Previous statistics show higher (66.21%) (12) and lower (21.64%) (10) positive rates of respiratory pathogens in other regions, which may be attributed to the differences in tested pathogen spectrum and study populations. Most epidemiological studies center on the detection of respiratory viruses, acute respiratory infections, and RTIs in the pediatric population. Our study comprehensively analyzed the most common respiratory pathogens, including respiratory viruses and MP, and recruited individuals across all age groups. Inconsistent with other regions, RTIs in Changzhou were predominantly caused by HRV, ADV, and MP, suggesting that the dominant respiratory pathogen spectrum varies by region. RTIs show distinct epidemiological characteristics in regions with different geographical and climatic features and testing methods adopted by local institutions (11, 13).

In our study, HRV was the most prevalent pathogen leading to RTIs in Changzhou from December 2023 to November 2025. Co-infections are common in RTIs (12–15). HRV combined with other respiratory pathogens was the leading cause of co-infections in our medical institution. The dominant pathogen responsible for RTIs, however, varies in China. A nationwide study demonstrated that FluA (28.5%) and *Streptococcus pneumoniae* (29.9%) are the most frequent bacterial viruses causing RTIs (16). Li et al. (11) reported that FluB is the most prevalent respiratory pathogen in Zhuhai (Guangdong, China) in 2010. In our local hospital, 1,659 individuals were infected by two or more respiratory pathogens during the two-year period. The co-infection rate was 6.80%, approaching to the positive rate reported by Xu et al. (5) (4.38%), but lower than that reported by Shen et al. (12) (20.87%). Pathogen interference potentially influences the positivity for co-infections of respiratory viruses or bacteria, though the precise mechanisms remain unclear (17). However, some studies have suggested that the interference may be due to the resource competition, the immune response, or interference through viral proteins (18, 19).

In the present study, females (48.47%) were more affected by RTIs than males (45.78%), showing a sex preference in the two time periods (2023–2024, and 2024–2025) and emphasizing sex-specific prevention and treatment strategies. Jiang et al. (9) consistently reported a significantly higher positive rate of common respiratory viruses in women than men. However, previous studies demonstrated a comparable detection rate of RTIs between males and females (16, 20). In our study, the positivity for single infections of FluA, FluB, ADV, and RSV, as well as co-infections with two or three respiratory pathogens did not differ significantly between the two sex groups. Inconsistent findings about the sex preference in RTIs can be attributed to hormonal influences, vaccination status, lifestyle, and healthcare-seeking behaviors (21–23).

Respiratory pathogens are typically seasonal (24). We consistently illustrated an obvious seasonal trend of respiratory pathogens in Changzhou. From December 2023 to November 2025, FluA surged in winter and autumn, though its seasonal trend differs in southern China (11, 16) and the United States (25), indicating regional variations in the circulation of respiratory pathogens. HRV displays multi-seasonal circulation, with distinct peaks in autumns and springs. In contrast, other respiratory pathogens we tested showed sporadic activities without clear seasonal trends, which is inconsistent with statistics reported at home and abroad (9, 26). In our study, respiratory pathogens spiked in winter during 2023–2024 and autumn during 2024–2025. A humid subtropical climate in Changzhou largely determines the distinct seasonal trend of respiratory pathogens. Cold and humid winters and autumns, and huge temperature variations in indoor and outdoor environments breed the seasonality of respiratory pathogens in Changzhou. Therefore, seasonal prevention strategies against RTIs are necessary in Changzhou.

Across all age groups, Flu A and B infections mainly affected teenagers (11.97%) and young adults (6.83%), respectively. Children were the most vulnerable to ADV (24.14%) and MP infections (27.48%). Infants (22.35%) and preschool children (21.36%) were more likely to be affected by RSV and HRV, respectively. Overall, the prevalence of RTIs varies by age. FluA posed the highest threat to teenagers at 12–17 years who are frequently engaged in school settings where the virus transmission is accelerated. Young adults were more prone to FluB infection due to the increased social activities. ADV, the second leading respiratory pathogen in Changzhou, predominantly affected children at 6–11 years, which is inconsistent with previous findings that ADV is the most common respiratory virus in childhood (27, 28). MP also predominantly affected school-aged children who are highly vulnerable to RTIs due to their long-term contact in high-transmission environments and less-developed immune systems (11). This age group is a high-risk population requiring targeted preventions. Consistent with previous research, RSV had the greatest impact on infants (12, 27, 29), showing an age-specific feature. Across all age groups, HRV (13.27%) presented the highest positive rate among the six detected respiratory pathogens, peaking in preschool children aged 3–5 years. Both HRV and RSV were the most common respiratory pathogens attacking children under five years, consistently supporting the global trend of RTIs in the pediatric population still in immune development and at high risks of viral infections (29).

Inconsistent with the data reported by Li et al. (16), the overall detection rate of RTIs was found significantly higher in outpatients than inpatients in our local hospital during the two time periods. This epidemiological pattern could be explained by non-pharmaceutical interventions (e.g., mask-wearing, personal hygiene) after the COVID-19 pandemic that remarkably curtail the transmission of respiratory pathogens in inpatients (22). Additionally, RT-PCR testing was performed when outpatients presented multiple symptoms of RTIs, such as fever, cough, nasal obstruction, sneezing, and sore throat. In our study, some patients were hospitalized not for RTIs. RT-PCR testing was conducted when they showed symptoms of suspected RTIs or not. Lastly, this epidemiological pattern may be attributed to the differences in healthcare-seeking behaviors between inpatients and outpatients. These factors will be introduced into future studies to verify our results. This highlighted comprehensive primary care strategies to prevent the occurrence or progression of RTIs to severe conditions requiring hospitalization. Importantly, this association may be reversed for RSV infection and MP infection as age was considered.

In our current study, we observed the effect of Simpson’s paradox. The OR for inpatients was reversed between the univariate and multivariate models for RSV infection and MP infection. Although univariate regression analysis showed that hospitalized patients were a protective factor, age, as a confounding variable, might have bring with bias. The median age of inpatients was 41 years, while that of outpatients was 6 years. In addition, the populations of RSV and MP were disproportionate among infants and young children. When using multiple regression to control for age, we revealed an ultimate causal relationship: hospitalization was a risk factor.

Several limitations were noted. First, we did not analyze the clinical characteristics of RTIs, including symptoms, imaging findings, vaccination status, and severity scores. Second, Flu subtypes, bacterial pathogens and other common viruses such as SARS-CoV-2 causing RTIs were not detected, thus providing an incomplete picture of the epidemiology of RTIs in Changzhou. Third, this was a single-center, retrospective study, leading to potential recall or selection biases. Lastly, a long-term observation was essential to expand the local spectrum of respiratory pathogens. Large-scale, multi-center prospective studies are warranted to depict a holistic view of respiratory pathogen epidemiology in Changzhou, and guide evidence-based control measures.

## 5 Conclusion

In this single-center, two-year, retrospective study of epidemiological characteristics of RTIs in Changzhou from December 2023 to November 2025, individuals across all age groups were examined by RT-PCR for six common respiratory pathogens. Single infections dominate, and HRV and ADV are considered as the leading respiratory pathogens. Sex, age, seasonality, and patient status were associated with epidemiological characteristics of RTIs in Changzhou. Our findings may provide valuable data for optimizing diagnostic and preventive strategies against RTIs in Changzhou, China.

## Data Availability

The original contributions presented in the study are included in the article/supplementary material, further inquiries can be directed to the corresponding authors.
